# Minnelide: A Novel Therapeutic That Promotes Apoptosis in Non-Small Cell Lung Carcinoma In Vivo

**DOI:** 10.1371/journal.pone.0077411

**Published:** 2013-10-15

**Authors:** Ilona Rousalova, Sulagna Banerjee, Veena Sangwan, Kristen Evenson, Joel A. McCauley, Robert Kratzke, Selwyn M. Vickers, Ashok Saluja, Jonathan D’Cunha

**Affiliations:** 1 Division of Basic and Translational Research, Department of Surgery, University of Minnesota, Minneapolis, Minnesota, United States of America; 2 Hematology, Oncology and Transplantation, University of Minnesota, Minneapolis, Minnesota, United States of America; 3 Department of Cardiothoracic Surgery, University of Pittsburgh, Pittsburgh, Pennsylvania, United States of America; French National Centre for Scientific Research, France

## Abstract

**Background:**

Minnelide, a pro-drug of triptolide, has recently emerged as a potent anticancer agent. The precise mechanisms of its cytotoxic effects remain unclear.

**Methods:**

Cell viability was studied using CCK8 assay. Cell proliferation was measured real-time on cultured cells using Electric Cell Substrate Impedence Sensing (ECIS). Apoptosis was assayed by Caspase activity on cultured lung cancer cells and TUNEL staining on tissue sections. Expression of pro-survival and anti-apoptotic genes (*HSP70*, *BIRC5, BIRC4, BIRC2, UACA, APAF-1*) was estimated by qRTPCR. Effect of Minnelide on proliferative cells in the tissue was estimated by Ki-67 staining of animal tissue sections.

**Results:**

In this study, we investigated *in* *vitro* and *in* *vivo* antitumor effects of triptolide/Minnelide in non-small cell lung carcinoma (NSCLC). Triptolide/Minnelide exhibited anti-proliferative effects and induced apoptosis in NSCLC cell lines and NSCLC mouse models. Triptolide/Minnelide significantly down-regulated the expression of pro-survival and anti-apoptotic genes (*HSP70*, *BIRC5, BIRC4, BIRC2, UACA*) and up-regulated pro-apoptotic *APAF-1* gene, in part, via attenuating the NF-κB signaling activity.

**Conclusion:**

In conclusion, our results provide supporting mechanistic evidence for Minnelide as a potential in NSCLC.

## Introduction

Lung cancer is the leading cause of cancer-related mortality in the US [1]. It has been estimated that 228,190 new cases and 159,480 deaths from lung cancer (NSCLC and SCLC (small cell lung carcinoma) combined) will occur in the US in 2013 [1]. NSCLC is the major subtype of lung cancer and represents approximately 85% of all cases. Almost 70% of lung cancer patients present with locally advanced or metastatic disease (stage III-IV) at the time of diagnosis. Despite the large number of clinical trials and considerable progress in the treatment during the past decade, the 5-year relative survival rate remains dismal, varying from 2% to 16% for these patients [2]. Therefore, development of novel anticancer agents in NSCLC is urgently needed to improve the outcome of therapy. 

Triptolide, a diterpenoid triepoxide, is a major bioactive component of the Chinese herb Tripterygium wilfordii Hook F or Thunder God Vine. Triptolide was purified from the roots of this plant in 1972 [3] and it possesses a broad-spectrum therapeutic properties, mainly anti-inflammatory, immunosuppressive, and anti-tumor activities [4]. Its cytotoxic effect was demonstrated in a wide variety of epithelial and hematological cancer cell lines, including pancreatic [5-8], gastric [9], colorectal cancer cells [10], as well as in neuroblastoma [11-13], and NSCLC cells [14-17]. Since triptolide is a hydrophobic agent and it cannot be used clinically, we synthetized its water-soluble pro-drug called Minnelide [18]. In preclinical studies, Minnelide was evaluated as a potent chemotherapeutic agent against pancreatic cancer [18] and osteosarcoma [19]. The precise mechanism of how triptolide/Minnelide kills cancer cells is not known. We, and others, have previously shown that triptolide decreased expression of heat shock proteins through down-regulation of NF-κB pathway [11,20-22].

Recent studies with cell culture systems and animal models have proposed the complex pathogenic role of NF-κB in lung cancer carcinogenesis [23-27]. NF-κB can be activated by several different mechanisms in lung cancer and pre-neoplastic lesions driven by different oncogenes, carcinogens, mediators of inflammation and/or other mechanisms such as the crosstalk between NF-κB and the PI3K/Akt/mTOR pathway [28]. In lung adenocarcinomas, an IKK-mediated activation of NF-κB via the phosphorylation of FADD is associated with poor prognosis [29]. Moreover, EGF-induced phosphorylation at tyrosine residue 42 in IκBα leads to IKK-independent NF-κB activation in lung adenocarcinomas [30]. A constitutively activated NF-κB pathway is related to the resistance to chemotherapy and radiotherapy in lung cancer [31,32]. Previous *in vitro* studies have documented that triptolide blocks trans-activation of p65 and thus sensitizes NSCLC cells to TRAIL-induced apoptosis [14,15]. It has also been shown that induction of apoptosis by Apo2L/TRAIL in NSCLC cells requires activation of extracellular signal-regulated kinase 2 (ERK2) [16]. Moreover, in these cells, triptolide blocks TNF (tumor necrosis factor)-α-induced expression of cIAP (cellular inhibitor of apoptosis protein)-1 and -2 proteins through inhibition of NF-κB activation [14]. Furthermore, triptolide induces the expression of HIF-1α (hypoxia-inducible factor-1α) protein, but suppress its transcriptional activity indicated by lowered secretion of vascular endothelial growth factor protein [17]. Another study found that triptolide can inhibit TNF-α-induced COX-2 expression by modulation of mRNA stability and post-translational regulation in A549 cells [33]. 

Inhibitor of apoptosis proteins (IAPs) are a group of anti-apoptosis proteins which serve as endogenous inhibitors of apoptotic cell death. Some IAPs, such as XIAP (X-chromosome linked inhibitor of apoptosis protein) and cIAP1 can directly inhibit certain initiator and effector apoptotic caspases [34,35]. Currently, there are eight known members of the IAP family, including NAIP (BIRC1), c-IAP1 (BIRC2), c-IAP2 (BIRC3), XIAP (BIRC4), survivin (BIRC5), Apollon/Bruce (BIRC6), ML-IAP (BIRC7 or livin) and ILP-2 (BIRC8). The overexpression of IAPs in tumors has also been associated with resistance to therapy and shorter overall patient survival [36]. IAPs mediate pro-survival signals due to activation of NF-κB and/or MAPK signaling pathways. Moreover, c-IAP1 and c-IAP2 are negative regulators of non-canonical NF-κB signaling through their ability to suppress cellular NIK levels [37-39]. Furthermore, overexpression of XIAP and survivin contribute to radio- and chemoresistance of NSCLCs [40-43]. 

Apaf-1 (apoptotic protease activating factor 1) protein is a core component of the apoptosome complexes, which are inducibly assembled in the cytosol of apoptosis committed cells. These heptameric complexes are formed after activation of Apaf-1 monomers by (d)ATP and holocytochrome-c, which had been released from mitochondria upon permeabilization of their outer membrane, and recruit and activate procaspase-9 molecules [44-46]. The active apoptosome-associated caspase-9 molecules the cleave and activate the zymogens of the effector caspases -3 and -7, which proteolytically target many intracellular proteins producing loss-of-function or gain-of-function fragments [47] and consequently bring about apoptosis execution of cells with caspase activity exceeding the apoptosis threshold [48,49]. 

There is evidence that impairment in apoptosome pathway signaling significantly contributes to chemo- and radioresistance in different types of tumor cells including NSCLC cells [50-58]. Recently, it has been discovered that Apaf-1 also has a non-apoptotic function in the DNA repair process, after being translocated from the cytoplasm into the nucleus during proapoptotic stress [59]. The mechanism of Apaf-1 entry into the cell nucleus is currently not known. However, there is some evidence that the uveal autoantigen with coiled-coil domains and ankyrin repeats (UACA)/nucling may be involved in this translocation [60]. It has been shown that UACA/nucling is associated with canonical NF-κ signaling pathway [61-63] and was described as a functional regulator of sensitivity to extracellular inducers of apoptosis in cancer cells [64]. 

In the present study, we have investigated the therapeutic effect of Minnelide on murine models of human NSCLC tumor. Here, we demonstrate that Minnelide suppresses tumor growth in animal models. Furthermore, we show that triptolide induces apoptosis in NSCLC cells by concurrent inhibition of expression of several pro-survival genes, via down-regulation of their NF-κB-mediated transcriptional activation, and up-regulation of expression of several pro-apoptotic genes together with induction of apoptotic signaling. Our results provide evidence for therapeutic potential of Minnelide as an anticancer drug in NSCLC. 

## Materials and Methods

### Ethics Statement

All procedures were approved by the University of Minnesota Institutional Animal Care and Use Committee (IACUC).

### Cell lines and reagents

Human primary A549 cells adenocarcinoma and large cell lung carcinoma NCI-H460 cells were purchased from American Type Culture Collection (Manassas, VA, USA). Cells were cultured in RPMI-1640 Medium with 2.5 mM glutamine supplemented by 10% fetal bovine serum and 1% penicillin-streptomycin solution. All cell lines were cultured in humidified atmosphere with 5% CO2 at 37°C. Every two days, cell growth media were replaced with the fresh media. A549 and NCI-H460 cells were authenticated by STR profiling (GRCF, Johns Hopkins, Baltimore, MD, USA). 

### Triptolide treatment

Triptolide (Calbiochem, EMD Chemicals, Inc., Gibstown, NJ, USA) was dissolved in dimethyl sulfoxide (DMSO; Sigma-Aldrich, St. Louis, MO, USA) to a stock concentration of 1 mg/mL and aliquots were kept at -20°C. For cell viability, caspase assays and RNA extraction, cells were seeded in serum-containing media. Following of 24 hours incubation, cells were treated with varying concentration of triptolide in serum-free media for defined times at 37°C. Cells treated with equal dilutions of DMSO alone in serum-free served as a control.

### Cell Counting Kit-8 (CCK8) assay

Cell viability was performed using the Dojindo Cell Counting Kit-8 (Kumamoto, Japan). Cells were seeded into a 96-plate at 1x10^3^ cells per well and allowed to adhere overnight. After treatment with triptolide at various concentrations for 24, 48, and 72 h, 10 µl of the tetrazolium substrate was added to each well of the plate. Plates were incubated at 37 °C for 1 h, then the optical density (OD) was measured at 450 nm using a microplate reader (F-2500 Fluorescence Spectrophotometer, HITACHI). The cell inhibitory rate was calculated according to the following equation: the cell inhibitory rate = [1-(OD experiment – OD blank)/(OD control - OD blank)] x 100%. All experiments were done in triplicate and repeated three independent times.

### Caspase activity assay

Caspase-3/7 and caspase-9 activities were analyzed by the Caspase-Glo luminescence based assays (Promega, Madison, WI, USA) according to the manufacturer’s protocol. Cells were seeded and treated with triptolide as described above. Then 100 µL of appropriate Caspase-Glo reagents were added to each well. Caspase activity was normalized to the corresponding cell viability measurements.

### Electric cell-substrate impedance sensing (ECIS®) assay

ECIS experiments were conducted using an Applied Biophysics Model ECISZθ instrument (Applied Biophysics, Troy, NY, USA). Briefly, cells (7x10^4^/well) plated on collagen type I coated 8W10E+ electrode arrays (Applied Biophysics, Troy, NY, USA) adhered and allowed to formed a monolayer for 5 hours. After washing with PBS, cells were treated with 100 nM of triptolide and the resistance and capacitance were measured at 6400 Hz every 10 minutes for a period of 48 hours. During the experiments, cultures were maintained at 37°C and 5% CO^2^ in air.

### Flow cytometry detection of apoptosis, DNA damage and cell proliferation

Cells were seeded in 6-well plates and treated with triptolide in concentration of 100 or 200 nM for 24 and 48 hours. Apoptosis, DNA damage and cell proliferation assay was performed according manufacturer’s conditions and cells (10,000 events) were acquired by FACS Canto II Flow Cytometer and data were analyzed by Diva software (BD Biosciences, San Jose, CA, USA).

### Human xenograft lung cancer tumor model

All of the mouse experiments were approved by the Institutional Animal Care and Use Committee of the University of Minnesota and were performed according to institutional and national guidelines. Four- to 6-week old nude mice (nu/nu; NCI) were injected with 5x10^6^ A549 (or NCI-H460) cells in 0.1 mL of Matrigel (BD Biosciences, San Jose, CA, USA) into the right flank using a 24-gauge needle (day 1). Mice were randomized into treatment and control groups immediately after cell injections. Five days after tumor injection, mice began receiving daily intraperitoneal injections of a volume of 0.1 mg/mL Minnelide that achieves a final dose of 0.42 mg/kg of mouse weight [18,19]. Control animals were injected with the equivalent volume of phosphate-buffered saline (PBS) that would mirror the volume of Minnelide per dose. There were ten mice per treatment group. The tumor sizes were measured with a digital caliper (Fisher, Pittsburgh, PA, USA) and tumor volume (mm^3^) was determined as V = short diameter^2^ x long diameter x 0.5. Mice were sacrificed on the day 28 following tumor injection. Tumors were excised and weighed.

### Immunohistochemistry staining

Rabbit monoclonal antibody against Ki-67 (SP6; MA5-14520) were purchased from Thermo Scientific (Rockford, IL, USA). Formalin-fixed paraffin-embedded (FFPE) tissue sections (4 µm) were deparaffinized with xylene and rehydrated. Antigen retrieval was achieved by heating the slides in a steamer for 30 min with Reavel Decloaker (BioCare Medical, Concord, CA, USA) and then cooled for at least 30 min. To suppress the reaction of endogenous peroxidase within the tissues, the samples were treated with a Protein Block Serum-Free solution (Dako, Carpinteria, CA, USA) for 10 min. Slides were then placed in a humid chamber and incubated overnight with the primary Ki-67 at a dilution of 1:200. After washing with PBS, endogenous peroxidase activity was quenched with 3% hydrogen peroxide in PBS for 10 min followed by a 30-min incubation with peroxidase-conjugated secondary antibodies (MM620H and RMR622 H, respectively, BioCare Medical, Concord, CA, USA) at room temperature. Finally, slides were incubated with diaminobenzidine chromogen solution (Vector Laboratories, Inc., Burlingame, CA, USA) and counterstained with Mayer’s hematoxylin (Vector Laboratories, Inc., Burlingame, CA, USA). Negative controls were processed as above without the primary antibody. Ki-67 staining for A549 cells was detected by immunofluorescence using donkey anti-rabbit-AF488 antibody (Life Technologies, Grand Island, NY, USA). Apoptosis in tumor tissues was detected by a terminal deoxynucleotidyl transferase dUTP nick-end labeling (TUNEL) assay using an In Situ Cell Death Detection Kit (Roche, Mannheim, Germany) according to the manufacturer’s instruction. Expression of Ki-67 and TUNEL staining was analyzed using ImageJ software (Research Service Brand, National Institute of Mental Health, Maryland, USA). 

### Dual-Luciferase reporter assay for NF-κB

For transfections, cells were plated at a density of 2.5x10^4^ cells per well in twenty four-well plates. After 24 hours cells were transiently transfected with a total of 100 ng of plasmid DNA using the Attractene Transfection Reagen (Qiagen, Germantown, MD, USA) according to the manufacturer’s instructions. Twenty four hours after transfection, the cells were treated with TNF-α (20 ng/ml per well) or with TNF-α and 100 nM of triptolide for another 24 hours and harvested for luciferase assay. Luciferase activity was measured using the Dual-Luciferase Reporter Assay System (Promega, Madison, WI, USA) and TD-20/20 luminometer (Turner BioSystems, Sunnyvale, CA, USA). Fireﬂy luciferase activity from the NF-κB reporter was normalized against the Renilla luciferase activity of the control plasmid. 

### RNA purification and cDNA preparation

Total RNA was isolated from cells using the TRIzol Reagent (Life Technologies, Grand Island, NY, USA) according to the manufacturer's instructions. The concentration of total RNA was determined by the fotometric assay in Synergy 2 (BioTek Instruments, Inc., Winooski, VT, USA), and all isolated RNA samples had an A260 nm/A280 nm ratio >1.8. Total RNA was stored at -80°C. One µg of total RNA was reversely transcribed using High Capacity cDNA Reverse Transcription Kit (Life Technologies, Grand Island, NY, USA) on the Peltier Thermal Cycler 200 (MJ Research, Watertown, MA, USA) under following conditions: 25°C for 20 min, 48°C for 40 min, 95°C for 5 min, 25°C for 10 min. The cDNA was diluted 1:1 for use in real-time PCR and stored at -20°C.

### Real-time RT-PCR

The sequences of the oligonucleotide primers used in real-time RT-PCR assays of expression of the investigated transcripts are listed in Table 1, except HSF1 (heat shock factor 1) (Quantitect Primer Assay; Qiagen, Germantown, MD, USA). The primers and probes were designed using the program NCBI Pick Primers and were synthesized at Invitrogen^TM^ Custom DNA Oligos (Life Technologies, Grand Island, NY, USA). The real-time RT-PCR assays were run in duplicates in MicroAmp Optical 96-well reaction plates on the ABI 7300 Real Time PCR System (Life Technologies, Grand Island, NY, USA) using the QuantiTech SYBR green PCR System (Qiagen, Germantown, MD, USA). The RT-PCR reaction mixtures of a total volume of 25 μl contained 12.5 µl of QuantiTect SYBR green PCR system, 400 nM of forward and reverse primers, and 2.5 µl cDNA. Cycling was performed using the default conditions of the Sequence Detection System software version 1.3 (Life Technologies, Grand Island, NY, USA) 15 min at 95°C, followed by 40 rounds of 15 sec at 95°C and 1 min at 58°C. All data were normalized to the reference gene 18S (Quantitect Primer Assay; Qiagen, Germantown, MD, USA) as described previously [65].

**Table 1:** Primers used for real-time RT-PCR quantification of expression of the investigated transcripts.

**Gene**	**Forward primer**	**Reverse primer**
*HSP70*	5`-ACCAAGCAGACGCAGATCTTC-3`	5`-CGCCCTCGTACACCTGGAT-3`
*BIRC2*	5`-TGCTGGTGCATGCGTCGTCG-3`	5`-TACCCATGCACAAAACTACCTC-3`
*BIRC4*	5`-CCCTTGGACCGAGCCGATCG-3`	5`-AACCCTGCTCGTGCCAGTGTT-3`
*BIRC5*	5`-GACGACCCCATAGAGGAACATA-3`	5`-TTTCCTTTGCAATTTTGTTC-3`
*APAF-1*	5`-CCATCACAGCACCATCCA-3`	5`-ACATCACACCATGAACCCAAC-3`
*UACA*	5`-CATCCTTATACATGGAGTTGATATTACAA-3`	5`-TGTCCGCCCGTCTACATC-3`

### Statistical analysis

Data are presented as mean ± SEM of three separate experiments. The significance of the difference between the control and each individual experimental condition was analyzed by unpaired Student’s *t* test. A *P* ˂ 0.05 was considered as statistically significant difference.

## Results

### Triptolide decreases proliferation and viability of NSCLC cells

**Figure 1:**
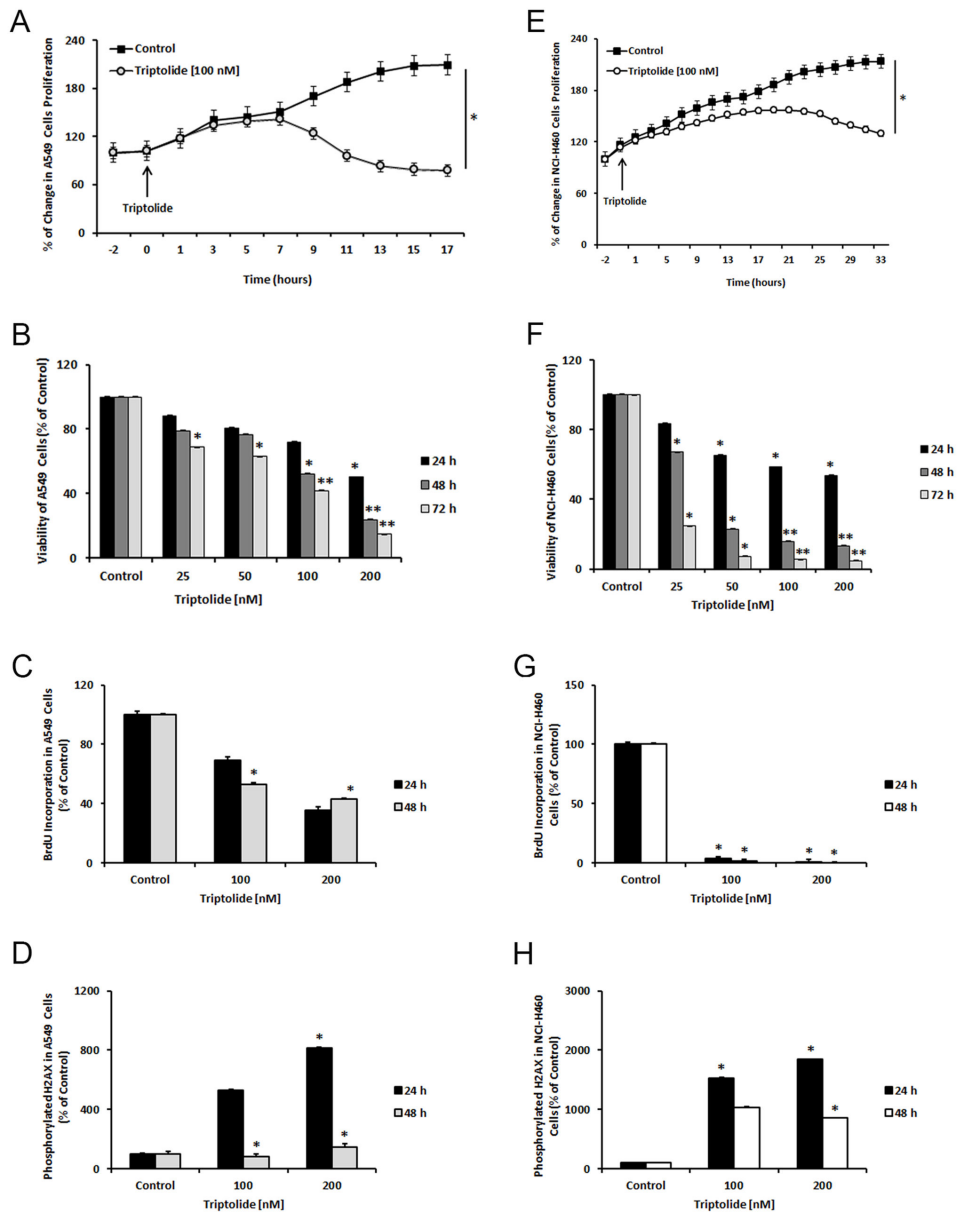
Triptolide decreases proliferation and viability of NSCLC cells. A549 cells (right panel) and NCI-H460 cells (left panel) were treated with 25-200 nM of triptolide for times indicated. Proliferation (A, E) and viability (B, F), as well as BrdU incorporation (C, G) were significantly reduced of both cell lines were reduced; however, the cytotoxic effect of triptolide was more pronounced in NCI-H460 cells. The indicator of DNA repair, phosphorylated H2AX, was significantly increased in both cell lines with triptolide treatment as shown (D, H). *Columns*, mean, *bars*, SE. Statistical significance of results was calculated with the Student`s *t* test (N=3) *P = 0.05; **P = 0.005.

Prior studies from our laboratory have shown that triptolide inhibited proliferation and viability in a variety of cancer cell lines. Similar to this, when A549 and NCI-H460 cells were treated with triptolide, growth was inhibited in a dose- and time-dependent manner (Figure 1 A and B, E and F). Effective growth inhibition, by 50%, was observed at doses of 100 and 200 nM after the 48-hour treatment. Viability of A549 and NCI-H460 cells decreased by 60-80% and 95% after the 72-hour treatment, respectively. Consistent with these observations, we next pursued multi-parameter flow cytometric analysis of triptolide treated A549 and NCI-H460 cells to gain an insight into the mechanism of cell death. We found that both cell lines treated with triptolide displayed significantly decreased levels of BrdU incorporation (Figure 1C and G), and simultaneously increased the level of phosphorylated H2AX (Figure 1D and H). These findings indicated reduced numbers of actively dividing cells in both cell lines as well as reduced DNA repair following triptolide treatment. 

### Triptolide induces apoptosis in NSCLC cells

**Figure 2:**
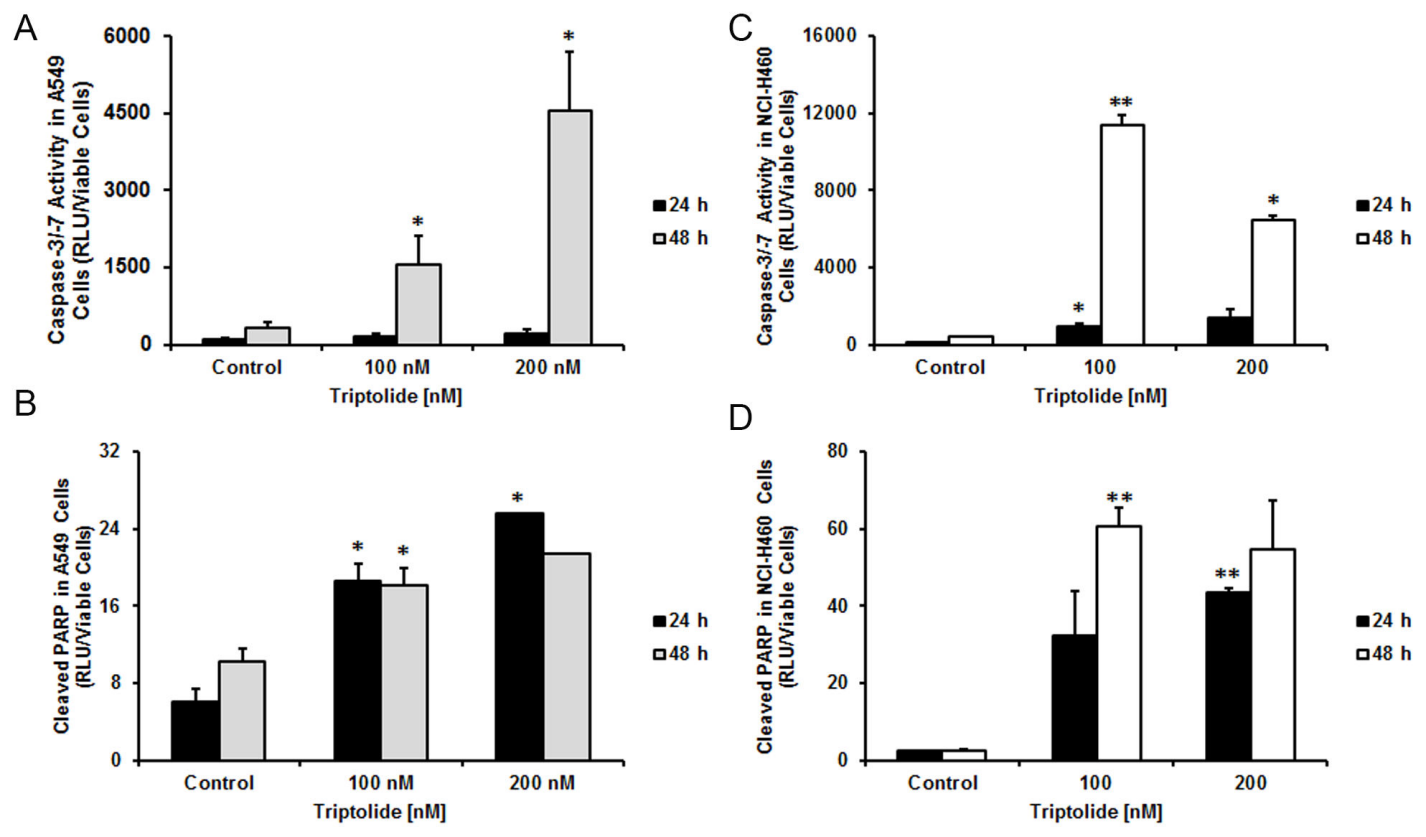
Triptolide induces apoptosis in NSCLC cells. NSCLC cells were exposed to 100 nM and 200 nM of triptolide for 24 and 48 hours and caspase-3/-7 activities (A, C) and PARP cleavage (B, D) were assessed. Following triptolide treatment caspase-3/-7 activity and cleavage of PARP were significantly increased compare to untreated cells demonstrating apoptosis. *Columns*, mean, *bars*, SE. Statistical significance of results was calculated with the Student`s *t* test (N=3) *P = 0.05; **P = 0.005. Results were expressed after normalizing to cell viability.

Since triptolide decreased proliferation, we next analyzed triptolide-treated cells for programmed cell death. We investigated two proteins involved in apoptosis, the activity of caspase-3/-7 and cleavage of PARP in A549 and NCI-H460 cell lines. Cells were treated with triptolide at two different concentrations (100 and 200 nM) for 24 and 48 hours for both experiments. In the triptolide-treated cells pro-caspase-3/-7 was significantly activated compared to the untreated cells (Figure 2A and C). During apoptosis, PARP (poly (ADP-ribose) polymerase) was cleaved and inactivated by caspase-3 that led to the inability of cells to repair DNA damage. Here, we show significantly increased levels of cleaved PARP in triptolide-treated cells in both cell lines (Figure 2B and D).

### Minnelide leads to tumor regression in xenograft mouse models

**Figure 3:**
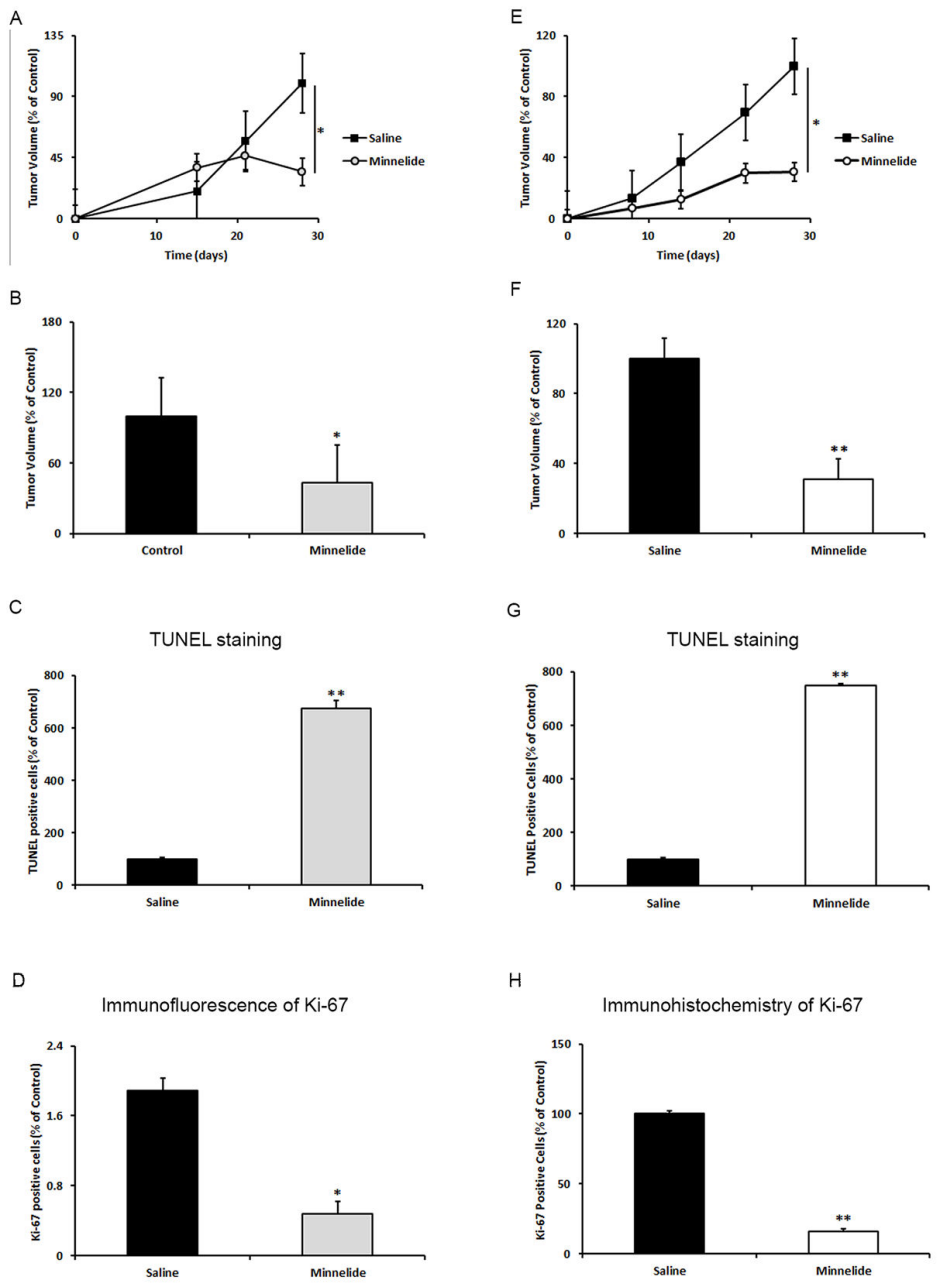
Minnelide leads to tumor regression in xenograft mouse models. In two xenograft mouse models, A549 (right panel) and NCI-H460 (left panel), tumor volumes were compared between minnelide treated (n = 10) and untreated groups (n = 10). Five days after tumor injection, mice began receiving daily intraperitoneal injections of minnelide at 0.42 mg/kg mouse weight. Control animals were injected with equivalent volumes of phosphate-buffered saline. Suppression of tumor growth occurred in the minnelide treated groups in comparison with the control groups in both xenograft models (A, E). Graphs (B and F) showed significantly decreased tumor volume from animals (A and E). Results were normalized to the untreated group for each cell line and expressed as the mean, *Columns*, *bars*, SE. Ki-67 protein expression was significantly decreased in the tumor tissue of minnelide treated group in both mouse models compare to saline treated groups (C and G). In concordance with decreased Ki-67 staining, TUNEL staining shown increased number of apoptotic cells in both mouse models (D and H). *Columns*, mean, *bars*, SE. Statistical significance of results was calculated with the Student`s *t* test (N=3) *P = 0.05; **P = 0.005.

To determine whether treatment with Minnelide effectively inhibited tumor growth *in vivo*, we established xenograft tumors in nude mice using A549 and NCI-H460 cell lines. Treatment with Minnelide resulted in a statistically significant reduction of tumor volume in both A549 and NCI-H460 xenograft models during the treatment period when compared with control groups, respectively (Figure 3, A and E). At the completion of experiments, tumor volume decreased in Minnelide treated animals, by an average of 2.9 times for A549 (*P* = 0.009) and 3.2 times for the NCI-H460 xenograft model (*P* = 0.001) (Figure 3, E and F). The actual tumor weight and volume measurements at the end of the experiment are tabulated in Table S2 and S3 for both the models. We did not observe overt toxic effects of the treatment as determined by physical examination and body weight measurement (data not shown). Furthermore, immunohistochemistry or immunofluorescence analysis of Ki-67 protein, a cellular marker for proliferation, confirmed a significant reduction of Ki-67 staining in the Minnelide-treated groups in both xenograft models (Figure 3, C and G, and Suplementary data Figure S1), as well as in KRAS-LSL mouse model (Fig. S2). In concordance, increased DNA fragmentation in Minnelide-treated groups was observed using TUNEL assay (Figure 3, D and H, and Fig. S1 and S2). These results indicated that the Minnelide treatment significantly slowed tumor growth *in vivo* by inducing apoptosis.

### Triptolide inhibits NF-κB signaling and its transcriptional target genes

**Figure 4:**
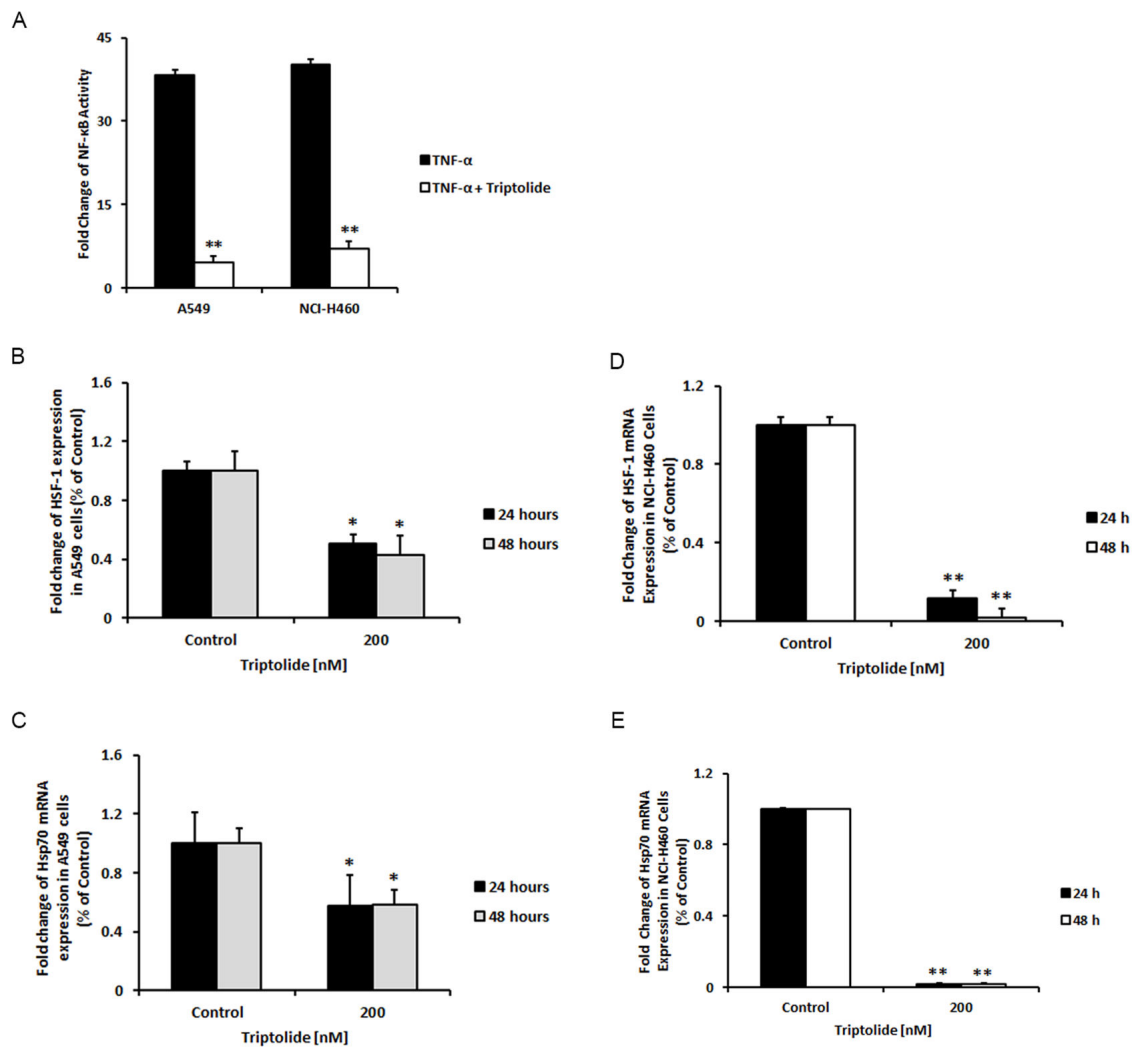
Triptolide inhibits NF-κB signaling pathway and its transcriptional activity of its target genes. NF-κB activity was assessed using dual-luciferase reporter assay after 24-hour treatment with TNF-α (20 ng/ml) or TNF-α (20 ng/ml) in combination with 100 nM of triptolide. The NF-κB activity was stimulated in the cells treated only with TNF-α, while it was effectively decreased after 100 nM of triptolide in A549 and NCI-H460 cells (A). Levels of HSF-1 and Hsp70 mRNA expressions were significantly down-regulated in A549 (B and C) and NCI-H460 cells (D and E). *Columns*, mean, *bars*, SE. Statistical significance of results was calculated with the Student`s *t* test (N=3) *P = 0.05; **P = 0.005.

NF-κB activation is an early and frequent phenomenon in the pathogenesis of lung cancer. To investigate the effect of triptolide on NF-κB activity in TNF-α-treated A549 and NCI-H460 cells, we exposed the cells to TNF-α alone (20 ng/ml) or TNF-α (20 ng/ml) in combination with triptolide [100 nM] for 24 hours and performed a dual-luciferase reporter assay. As shown in Figure 4A, NF-κB activity was significantly reduced after the treatment in both TNF-α-treated cell lines. Moreover, we analyzed several downstream transcripts of NF-κB signaling pathway, such as HSF-1 and HSP70 (heat shock protein 70). As expected, both genes were significantly down-regulated in both cell lines tested (Figure 4B and D, C and E).

### Triptolide induces cell death by down-regulating anti-apoptotic genes and enhances apoptosome formation

**Figure 5:**
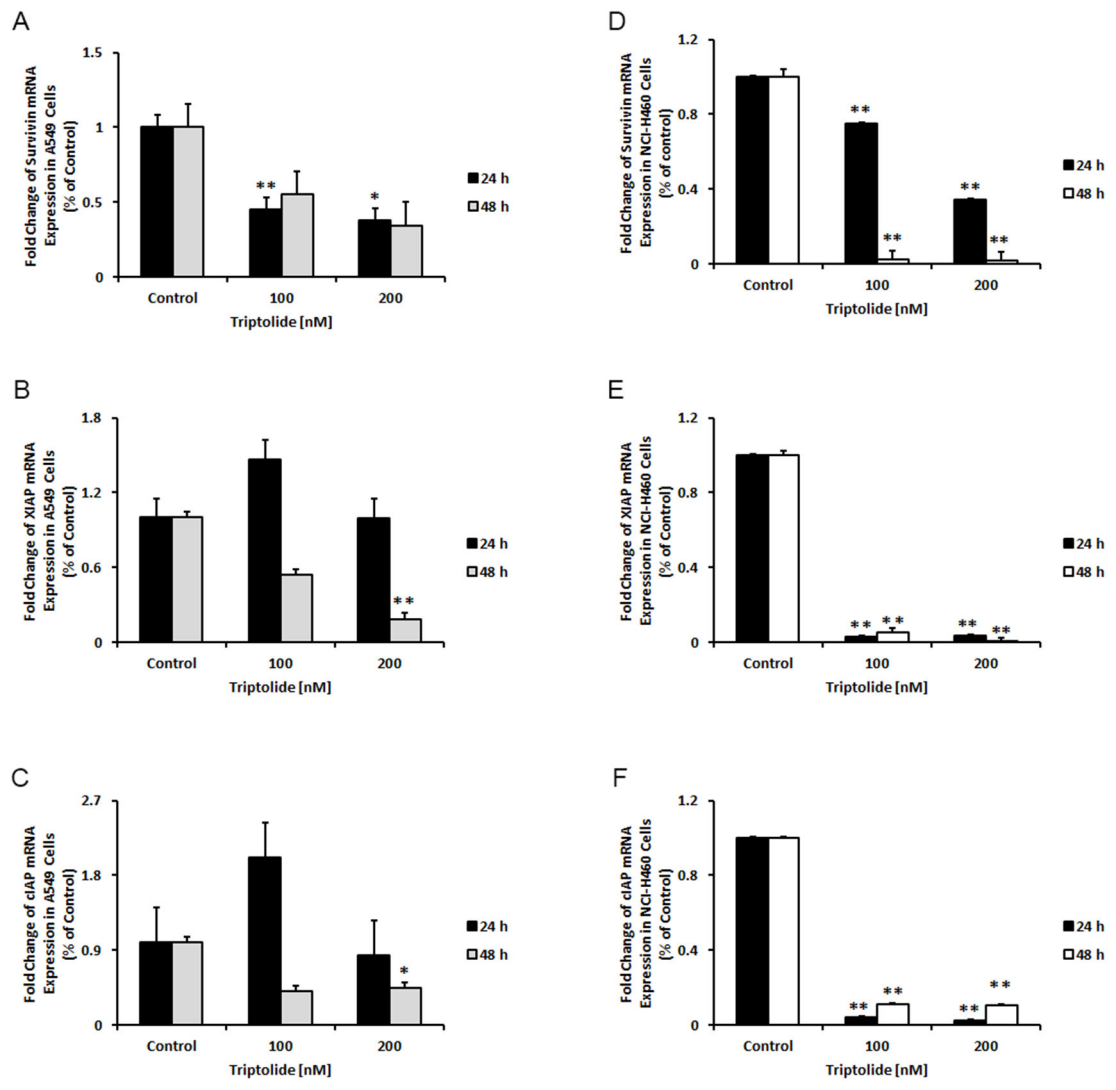
Triptolide down-regulates anti-apoptotic IAP genes in A549 and NCI-H460 cells. Triptolide significantly reduces the expression of IAP genes, (A and D) survivin, (B and E) XIAP and (C and F) c-IAP1. *Columns*, mean, *bars*, SE. Statistical significance of results was calculated with the Student`s *t* test (N=3) *P = 0.05; **P = 0.005.

**Figure 6:**
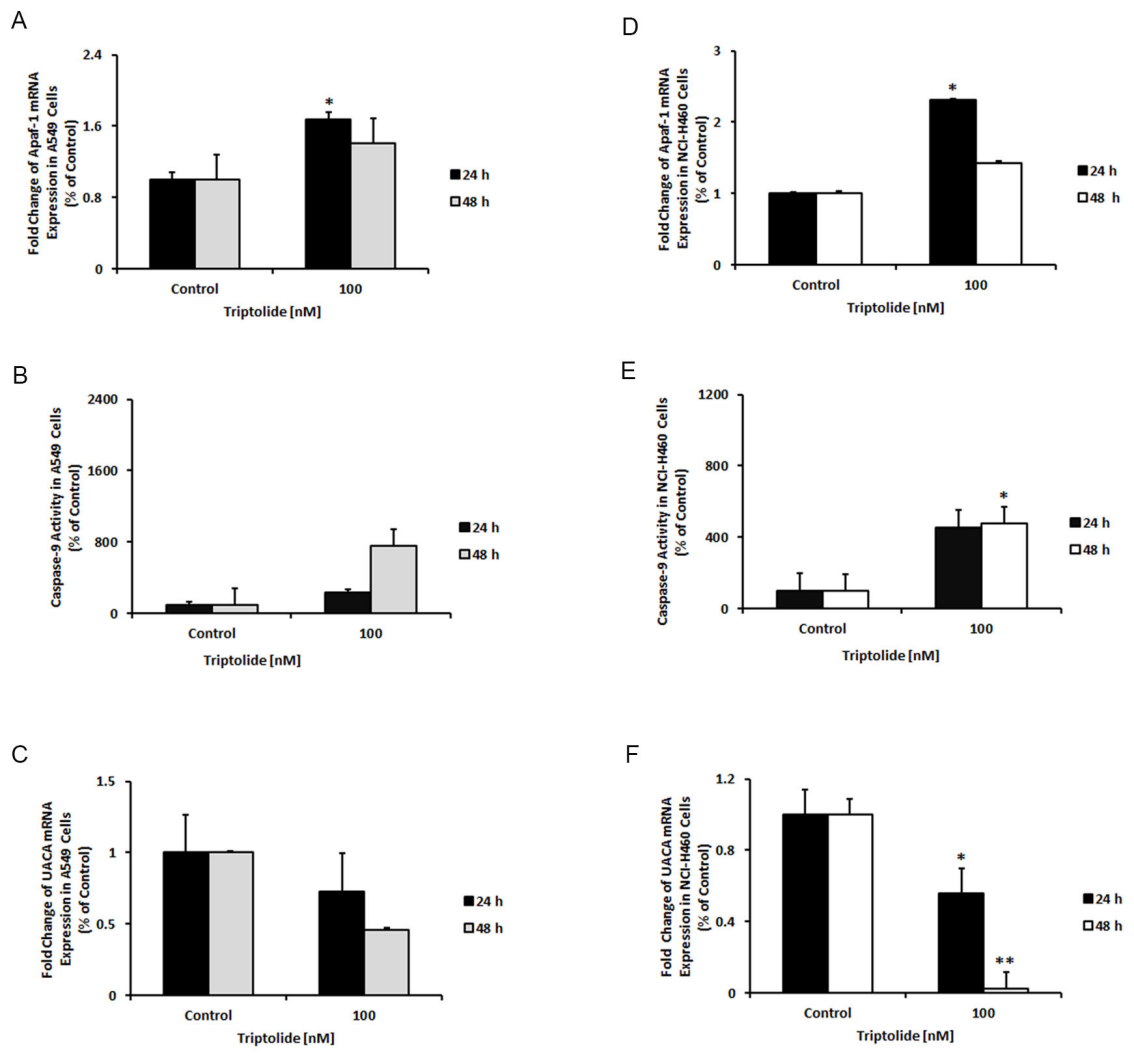
Triptolide promotes pro-apoptotic condition in A549 and NCI-H460 cells. Triptolide treatment increased Apaf-1 mRNA expression level in both NSCLC cell lines (A and D). In parallel, caspase-9 activity was increased in both NSCLC cell lines (B and E). Level of UACA mRNA expression was decreased after triptolide treatment (C and F). *Columns*, mean, *bars*, SE. Statistical significance of results was calculated with the Student`s *t* test (N=3) *P = 0.05; **P = 0.005.

The inhibitors of apoptosis proteins blocked apoptotic cell death through different mechanisms, including prevention of pro-caspase-9 activation and inhibition of the activity of caspases -9, -3 and -7. Here, we assessed the effect of triptolide on the expression level of survivin, XIAP and cIAP1 mRNAs. Our results showed significantly reduced levels of all these IAP transcripts in the triptolide-treated A549 and NCI-H460 cells (Figure 5). To investigate whether triptolide could promote the intrinsic apoptosome-mediated caspase activation pathway, we treated the cell lines with 100 nM of triptolide for 24 and 48 hours and subsequently analyzed the transcriptional level of Apaf-1 expression and activation of pro-caspase-9. Transcriptional expression of Apaf-1 was increased in A549 and NCI-H460 cells after the treatment (Figure 6A and D). To show that pro-caspase-9 is activated, we performed a luminescence assay. Triptolide treatment led to increased caspase-9 activity in both cell lines (Figure 6B and E). Recently, it has been discovered that Apaf-1 also has a non-apoptotic function in the DNA repair process, after being trans-located from the cytoplasm into the nucleus during apoptotic stress [55]. The mechanism of Apaf-1 entry into the nucleus [55] is not known to date. Since there is some evidence that UACA is responsible for the intranuclear Apaf-1 protein trans-location [56], we therefore tested the level of UACA mRNA expression under the same conditions as described above. Interestingly, the expression level of this transcript was significantly lower only in NCI-H460 cells (Figure 6C and F). 

## Discussion

NSCLC is an aggressive disease characterized by chemo- and radioresistance with marginal response rates of overall cure [66-68]. The majority of NSCLC patients are diagnosed with advanced disease and have a poor prognosis. Platinum-based chemotherapy is considered to be a standard therapy for patients with advanced disease and routinely only yields a 30% response rate, 4 months progression free survival with a median survival of 9-11 months with only 5% of patients alive at 2 years [69-71]. During the last few years, significant advances have been made in NSCLC treatment due to the discovery of activating EGFR (epidermal growth factor receptor) mutations as well as the introduction of TKI (tyrosine kinase inhibitors) in all lines of treatment. However, many EGFR TKI-sensitive patients subsequently develop acquired resistance to the treatment [72,73] with no effective therapeutic options. Several mechanisms of TKIs resistance have been described including secondary mutation in EGFR (T790M), overexpression of the MET receptor, activation of AXL kinase and/or the NF-κB pathway [74-79]. 

NF-κB signaling has recently been shown to be essential for KRAS driven tumor growth, chemoresistance and radioresistance in NSCLC [24,58,66-68]. In lung tumors, NF-κB is constitutively activated due to different changes in the tumor microenvironment, such as local inflammation, hypoxia, host immune responses, and smoking [25,26,80-82]. On the other hand, there is little evidence of constitutive activation of NF-κB in NSCLC cells [83]. Here, we showed that triptolide and its water-soluble analog Minnelide reduced cell proliferation (Figures 1 and 5) and induced programmed cell death in NSCLC cells (Figure 2) and xenograft mouse models (Figure 5). Furthermore, we demonstrated the inhibitory effect of triptolide on TNF-α-induced NF-κB activity in both A549 and NCI-H460 cells using the dual-luciferase reporter assay (Figure 4A). Since uncontrolled NF-κB expression negatively regulates apoptosis in tumor cells by affecting several anti-apoptotic genes, such as *HSF1*, *HSP70* and several members of the IAP family [8,12,84], we analyzed the effect of triptolide on the expression of several IAP genes. All investigated IAP transcripts were down-regulated in both cell lines by triptolide treatment (Figures 4 and 5), but this IAP repressing activity of triptolide was more pronounced in NCI-H460 cells compared to A549 cells. A possible explanation for the different response to the treatment might be different basal levels of NF-κB in these cells (data not shown) [83]. It is not surprising to us that different cell lines demonstrated these variabilities. These observations may also have important translational attributes as we explore why certain patients respond differently in the era of personalized treatment of NSCLC. 

Activation of the apoptosome apparatus, a stress-induced cell death-signaling platform, in the cytoplasm is an essential step for pro-caspase-9 activation. The initiation step for the apoptosome apparatus assembly is the binding of cytochrome-c to the functional Apaf-1 splice variants, Apaf-1LX and/or Apaf-1LC in the presence of dATP/ATP [85]. Once activated in apoptosomes, the apoptosome-associated caspase-9 cleaves and activates the down-stream effector pro-caspase-3 and -7 [44-46]. The active caspase-3 and -7 then proteolytically cleave other key intracellular regulatory and structural proteins [47]. Recently, a non-apoptotic role of intranuclear Apaf-1 has been proposed in the DNA damage checkpoint activation [59]. The exact mechanism of the Apaf-1 translocation into the nucleus has not been fully elucidated, so far. Sakai and colleagues [60] have shown some evidence that UACA/nucling could be the Apaf-1 nuclear translocator. We observed the up-regulation of Apaf-1 and down-regulation of UACA after the triptolide treatment of NSCLC cells (Figure 6). These findings together with the increased level of caspase-9 activity indicate that triptolide may promote the apoptosome-mediated apoptosis pathway through up-regulation of Apaf-1 in the cytoplasm. Moreover, suppression of UACA expression may contribute to retention of Apaf-1 protein in the cytoplasm and/or to translocation of glucose regulated protein 78 receptor to the cell surface and hence to sensitization of the cells to apoptosis by extracellular protease activated receptor-4 protein [64]. 

There is evidence that triptolide inhibits and sensitizes colorectal cancer cells and oral squamous cell carcinoma cells to 5-fluorouracil [10,86,87]. Additionally, triptolide also induces the cytotoxicity and enhanced carboplatin-mediated apoptosis in human ovarian cancer *in vitro* and *in vivo* [88]. It is important to note that both constitutive and inducible NF-κB activation by chemotherapeutics or radiation blunt their anticancer activities and that blocking NF-κB may circumvent the resistance caused, in part, by overexpression of anti-apoptotic genes, such as XIAP and survivin [42,89-91]. Here, we can hypothetize that triptolide/Minnelide might be classified as a NF-κB inhibitor and thus could sensitize lung cancer to chemotherapy [31,92], radiotherapy [93], and TKIs [79]. However, there is one caveat for using the inhibitors of NF-κB pathways since they might also suppress effective antitumor immunity [94].

## Conclusion

Our findings indicate that triptolide/Minnelide induced apoptosis in NSCLC cells and tumors. The mechanism of apoptosis is partially mediated by inhibition of NF-κB pathway, decreasing of anti-apoptotic genes, such as *HSP70*, *BIRC4*, *BIRC2* and *BIRC5*, and/or increasing pro-apoptotic *APAF-1* gene. Taken together, this study indicates a promising potential for the use of Minnelide as a future therapy for NSCLC patients. Our results provide a rationale for future clinical investigation of the therapeutic efficacy of Minnelide in NSCLC patients.

## Supporting Information

Methods S1 **Transgenic mouse model of spontaneous lung cancer.** For induction of spontaneous lung cancer, we used KRAS-LSL mouse model which utilizes the Cre-Lox system. To activate the oncogenic KRAS gene, Cre was delivered to the lung of mice via intranasal administration of Cre expressing Adenovirus (Ad-Cre) as described previously [95]. After 56 days, 5 animals were sacrificed and the tumor penetrance was 100%. The mice were randomized into the treated and control groups (N = 10). Following 28 days of Minnelide (0.42 mg/kg) or phosphate-buffered saline treatment, half of the mice in each group were sacrificed. The remaining animals (N = 5 in each group) were followed for a period of 28 days without treatment and then sacrificed and tumors assessed. All experiments involving animals were performed in accordance with the guidelines of the Institutional Animal Care and Use Committee of the University of Minnesota.
(DOCX)

Figure S1 **Immunohistochemistry staining of Ki-67 and TUNEL staining in xenograft mouse models.** Ki-67 protein expression was significantly decreased in the tumor tissue of Minnelide-treated group in xenograft A549 (A) and NCI-H460 (C) mouse models compare to saline treated groups (20x mag, scale 50 µm). TUNEL staining was significantly increased in xenograft A549 (A) and NCI-H460 (C) mouse models (20x mag, scale 50 µm) (B and D).
(TIF)

Figure S2 **Immunohistochemistry staining of Ki-67 and TUNEL staining in transgenic KRAS-LSL mouse model.** Ki-67 protein expression was significantly decreased in the tumor tissue of Minnelide-treated group in transgenic KRAS-LSL mouse models compare to saline treated groups (20x mag, scale 50 µm) (A). TUNEL staining was significantly increased in these mouse models (20x mag, scale 50 µm) (B). *Columns*, mean, *bars*, SE. Statistical significance of results was calculated with the Student`s *t* test (N=3) *P = 0.05; **P = 0.005.
(TIF)

Table S1 **Final tumor weight and final tumor volume in xenograft mouse model A549.**
(PDF)

Table S2 **Final tumor weight and final tumor volume in xenograft mouse model NCI-H460.**
(DOCX)

Table S3 **Final tumor weight and final tumor volume in xenograft mouse model NCI-H460.**
(DOCX)
