# Supplementary material for: Minnelide: A Novel Therapeutic That Promotes Apoptosis in Non-Small Cell Lung Carcinoma In Vivo
Source: PLoS One. 2013 Oct 15;8(10):e77411. doi: 10.1371/journal.pone.0077411 (PMC3797124; doi:10.1371/journal.pone.0077411)
Supplement: Methods S1 — Transgenic mouse model of spontaneous lung cancer. For induction of spontaneous lung cancer, we used KRAS-LSL mouse model which utilizes the Cre-Lox system. To activate the oncogenic KRAS gene, Cre was delivered to the lung of mice via intranasal administration of Cre expressing Adenovirus (Ad-Cre) as described previously [95]. After 56 days, 5 animals were sacrificed and the tumor penetrance was 100%. The mice were randomized into the treated and control groups (N = 10). Following 28 days of Minnelide (0.42 mg/kg) or phosphate-buffered saline treatment, half of the mice in each group were sacrificed. The remaining animals (N = 5 in each group) were followed for a period of 28 days without treatment and then sacrificed and tumors assessed. All experiments involving animals were performed in accordance with the guidelines of the Institutional Animal Care and Use Committee of the University of Minnesota. (DOCX) [file pone.0077411.s001.docx]

## Supporting information

**Transgenic mouse model of spontaneous lung cancer**. For induction of spontaneous lung cancer, we used KRAS-LSL mouse model which utilizes the Cre-Lox system. To activate the oncogenic *KRAS* gene, Cre was delivered to the lung of mice via intranasal administration of Cre expressing Adenovirus (Ad-Cre) as described previously [95]. After 56 days, 5 animals were sacrificed and the tumor penetrance was 100%. The mice were randomized into the treated and control groups (N = 10). Following 28 days of Minnelide (0.42 mg/kg) or phosphate-buffered saline treatment, half of the mice in each group were sacrificed. The remaining animals (N = 5 in each group) were followed for a period of 28 days without treatment and then sacrificed and tumors assessed. All experiments involving animals were performed in accordance with the guidelines of the Institutional Animal Care and Use Committee of the University of Minnesota.

**Figure S1 Immunohistochemistry staining of Ki-67 and TUNEL staining in xenograft mouse models.** Ki-67 protein expression was significantly decreased in the tumor tissue of Minnelide-treated group in xenograft A549 (A) and NCI-H460 (C) mouse models compare to saline treated groups (20x mag, scale 50 µm). TUNEL staining was significantly increased in xenograft A549 (A) and NCI-H460 (C) mouse models (20x mag, scale 50 µm) (B and D).

**Figure S2 Immunohistochemistry staining of Ki-67 and TUNEL staining in transgenic KRAS-LSL mouse model.** Ki-67 protein expression was significantly decreased in the tumor tissue of Minnelide-treated group in transgenic KRAS-LSL mouse models compare to saline treated groups (20x mag, scale 50 µm) (A). TUNEL staining was significantly increased in these mouse models (20x mag, scale 50 µm) (B). *Columns*, mean, *bars*, SE. Statistical significance of results was calculated with the Student`s *t* test (N=3) **P* = 0.05; ***P* = 0.005.
