# Supplementary figures and images for: Minnelide: A Novel Therapeutic That Promotes Apoptosis in Non-Small Cell Lung Carcinoma In Vivo
Source: PLoS One. 2013 Oct 15;8(10):e77411. doi: 10.1371/journal.pone.0077411 (PMC3797124; doi:10.1371/journal.pone.0077411)

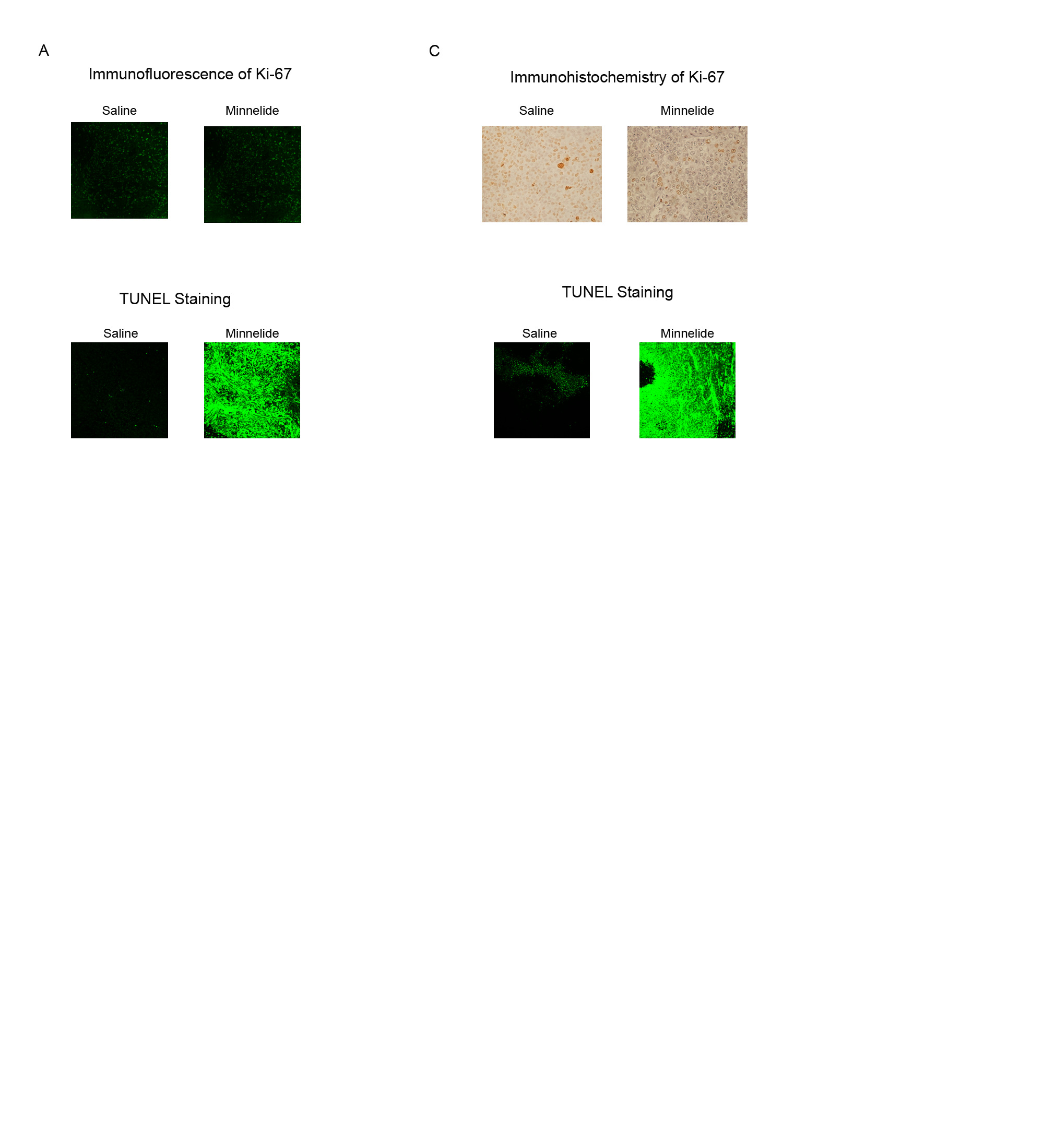

Supplement: Figure S1 — Immunohistochemistry staining of Ki-67 and TUNEL staining in xenograft mouse models. Ki-67 protein expression was significantly decreased in the tumor tissue of Minnelide-treated group in xenograft A549 (A) and NCI-H460 (C) mouse models compare to saline treated groups (20x mag, scale 50 µm). TUNEL staining was significantly increased in xenograft A549 (A) and NCI-H460 (C) mouse models (20x mag, scale 50 µm) (B and D). (TIF) [file pone.0077411.s002.tif]

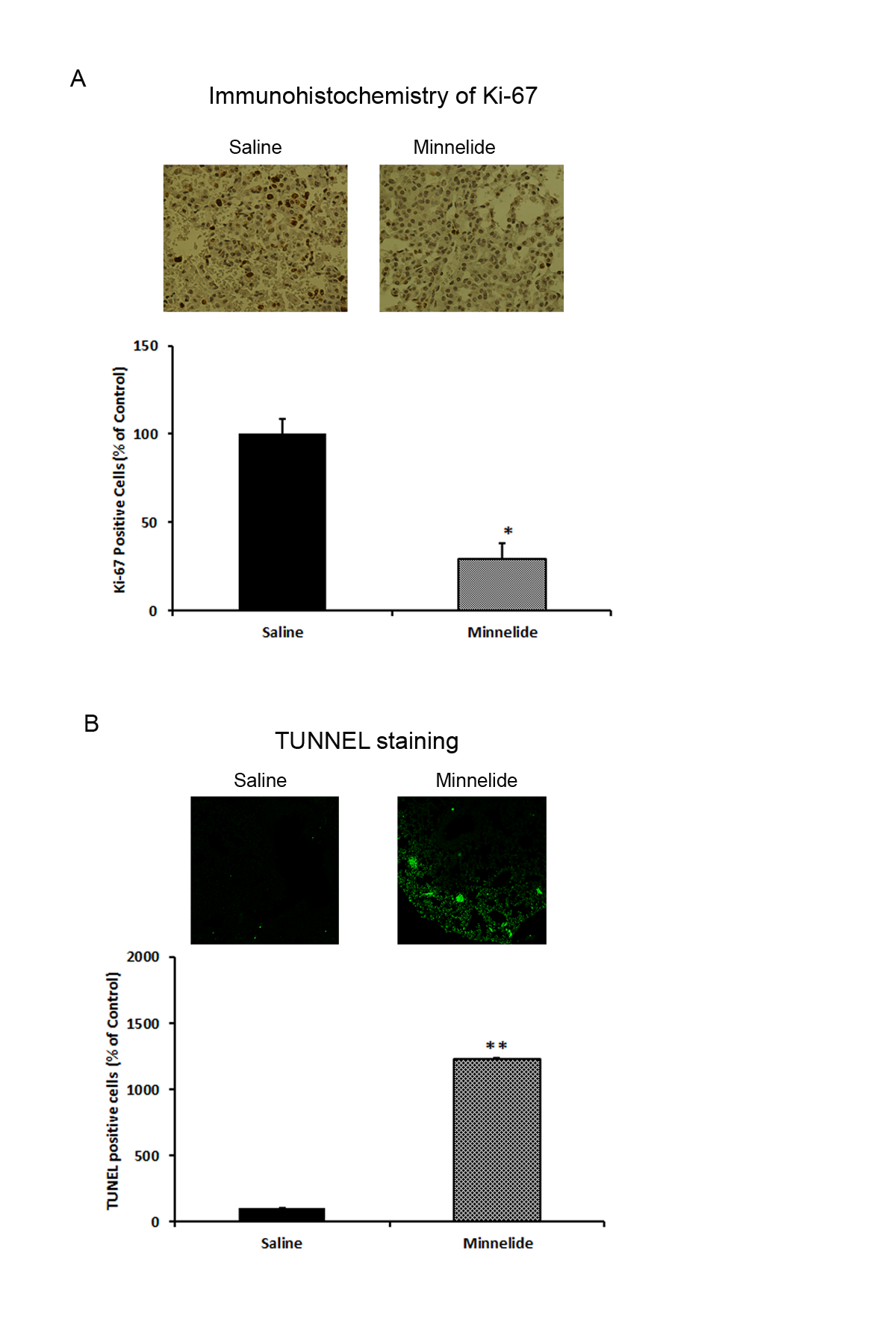

Supplement: Figure S2 — Immunohistochemistry staining of Ki-67 and TUNEL staining in transgenic KRAS-LSL mouse model. Ki-67 protein expression was significantly decreased in the tumor tissue of Minnelide-treated group in transgenic KRAS-LSL mouse models compare to saline treated groups (20x mag, scale 50 µm) (A). TUNEL staining was significantly increased in these mouse models (20x mag, scale 50 µm) (B). Columns, mean, bars, SE. Statistical significance of results was calculated with the Student`s t test (N=3) *P = 0.05; **P = 0.005. (TIF) [file pone.0077411.s003.tif]
