# Supplementary material for: Minnelide: A Novel Therapeutic That Promotes Apoptosis in Non-Small Cell Lung Carcinoma In Vivo
Source: PLoS One. 2013 Oct 15;8(10):e77411. doi: 10.1371/journal.pone.0077411 (PMC3797124; doi:10.1371/journal.pone.0077411)
Supplement: Table S1 — Final tumor weight and final tumor volume in xenograft mouse model A549. (PDF) [file pone.0077411.s004.pdf]

Supplementary Table I. Primers used for real-time RT-PCR quantification of expression of the investigated transcripts

Gene	Forward primer	Reverse primer
HSP70	5`-ACCAAGCAGACGCAGATCTTC-3`	5`-CGCCCTCGTACACCTGGAT-3`
BIRC2	5`-TGCTGGTGCATGCGTCGTCG-3`	5`-TACCCATGCACAAAACTACCTC-3`
BIRC4	5`-CCCTTGGACCGAGCCGATCG-3`	5`-AACCCTGCTCGTGCCAGTGTT-3`
BIRC5	5`-GACGACCCCATAGAGGAACATA-3`	5`-TTTCCTTTGCAATTTTGTTC-3`
APAF-1	5`-CCATCACAGCACCATCCA-3`	5`-ACATCACACCATGAACCCAAC-3`
UACA	5`-CATCCTTATACATGGAGTTGATATTACAA-3`	5`-TGTCCGCCCGTCTACATC-3`
