# Supplementary material for: Minnelide: A Novel Therapeutic That Promotes Apoptosis in Non-Small Cell Lung Carcinoma In Vivo
Source: PLoS One. 2013 Oct 15;8(10):e77411. doi: 10.1371/journal.pone.0077411 (PMC3797124; doi:10.1371/journal.pone.0077411)
Supplement: Table S3 — Final tumor weight and final tumor volume in xenograft mouse model NCI-H460. (DOCX) [file pone.0077411.s006.docx]

Table 3. Final tumor weight and final tumor volume in xenograft mouse model NCI-H460.

|  | **Xenograft Mouse Model NCI-H460** | |
| --- | --- | --- |
|  | **Saline** | **Minnelide** |
| Final Tumor Weight (g) | 1.9 | 0.8 |
| Final Tumor Volume (mm^3^) | 2791 | 859 |
